# Supplementary material for: The Association between Gut Microbiome and Pregnancy-Induced Hypertension: A Nested Case–Control Study
Source: Nutrients. 2022 Nov 1;14(21):4582. doi: 10.3390/nu14214582 (PMC9657571; doi:10.3390/nu14214582)
Supplement: Supplementary file 1 [file nutrients-14-04582-s001.zip › nutrients-1969896-supplementary.pdf]

Table S1. Basic characteristics of subjects in PE and GH group.

Variable	Control (n=35)	Case	
		GH (n=20)	PE (n=15)
Age (year)	31.26±3.94	29.95±3.53	33.73±4.01[#*]
Gestational age (week)	12.70±0.86	13.49±4.28	12.69±0.80
Race			
Han	33(94.3%)	19(95.0%)	15(100.0%)
Others	2(5.7%)	1(5.0%)	0(0.0%)
Occupation			
Professionals	9(25.7%)	6(30.0%)	2(13.3%)
Company employee	14(40.0%)	10(50.0%)	5(33.3%)
Others	12(34.3%)	4(20.0%)	8(53.3%)
Education			
Junior college and below	18(51.4%)	7(35.0%)	7(46.7%)
Undergraduate and above	17(48.6%)	13(65.0%)	8(53.3%)
Monthly income			
10000 and below	18(51.4%)	10(50.0%)	12(80.0%)
10000 and above	17(48.6%)	10(50.0%)	3(20.0%)

#: Means there was statistics difference between GH and PE group ($p < 0.05$).

*: Means there was statistics difference between subgroup and control group ($p < 0.05$).

Table S2. Clinical characteristics of subjects in PE and GH group.

Variable	Control (n=35)	Case	
		GH (n=20)	PE (n=15)
Early waist (cm)	79.47±7.01	80.49±7.56	84.65±10.58
Early BMI (kg/m2)	21.80±2.20	22.49±3.48	24.32±3.49*
Early SBP (mmHg)	114.77±9.54	124.95±7.55*	125.20±8.43*
Early DBP (mmHg)	73.49±8.46	80.65±7.10*	83.20±7.50*
HGB (g/L)	122.71±10.51	127.78±6.89	127.37±7.98
GLU (mmol/L)	4.52±0.39	4.68±0.55	4.76±0.34
ALB (g/L)	45.84±2.26	45.48±2.16	44.33±2.79
ALT (U/L)	17.57±10.54	21.08±18.48	24.95±13.07
AST (U/L)	18.46±5.48	20.24±7.99	22.82±8.40
CREA (umol/L)	43.23±8.16	43.15±6.28	45.35±8.83
UA (umol/L)	197.89±38.93	219.35±50.49	245.64±56.72*
UREA (mmol/L)	2.50±0.55	2.66±0.90	2.56±0.59
TG (mmol/L)	1.42±0.40	1.48±0.64	1.92±0.60*
TCHOL (mmol/L)	4.74±0.86	4.85±0.89	4.27±0.82
HDLCH (mmol/L)	1.89±0.39	2.06±0.41	1.68±0.38
LDLCH (mmol/L)	2.74±0.78	2.69±0.82	2.35±0.47
hsCRP (mg/L)	3.86±4.20	3.92±2.86	6.32±6.80
INS (mU/L)	19.19±31.74	36.88±46.85	56.49±74.45*
GGT (U/L)	13.57±5.79	19.62±17.59	25.86±15.88*

*: Means there was statistics difference between subgroup and control group ($p < 0.05$).

BMI, body mass index; SBP, systolic blood pressure; DBP, diastolic blood pressure; HGB, hemoglobin; ALB, albumin; ALT, glutamic pyruvic transaminase; AST, glutamic oxalacetic transaminase; CREA, creatinine; UA, uric acid; TG, triglyceride; TCHOL, total cholesterol; HDLCH, high density lipoprotein cholesterol; LDLCH, low density lipoprotein cholesterol; hsCRP, hypersensitive C-reactive protein; INS, insulin; GGT, glutamyltransferase.

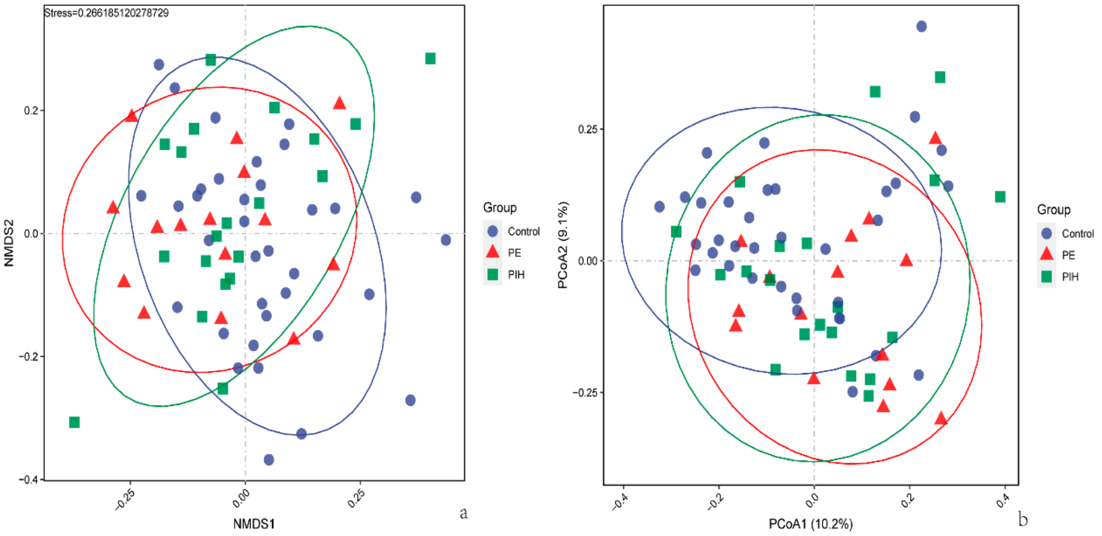

Figure S1. The beta diversity of subgroup and healthy controls. (a) NMDS analysis in three groups. (b) PCoA analysis in three groups.

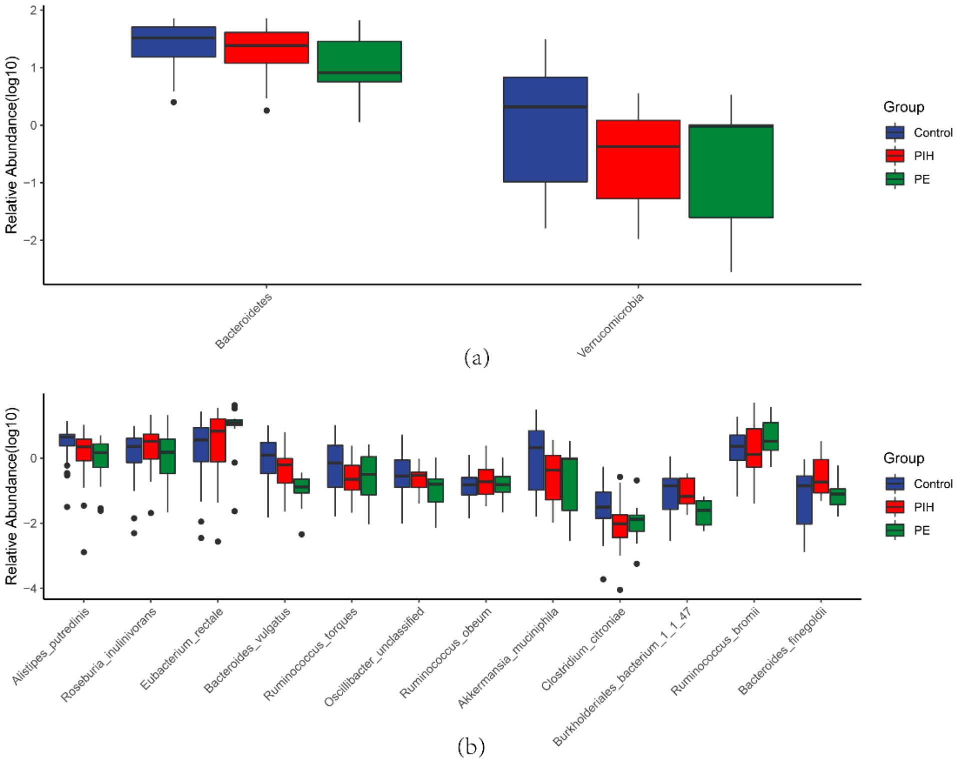

Figure S2. Boxplot of different microbial taxa. (A) Different phylum level taxa in three groups. (B) Different species level taxa in three groups.

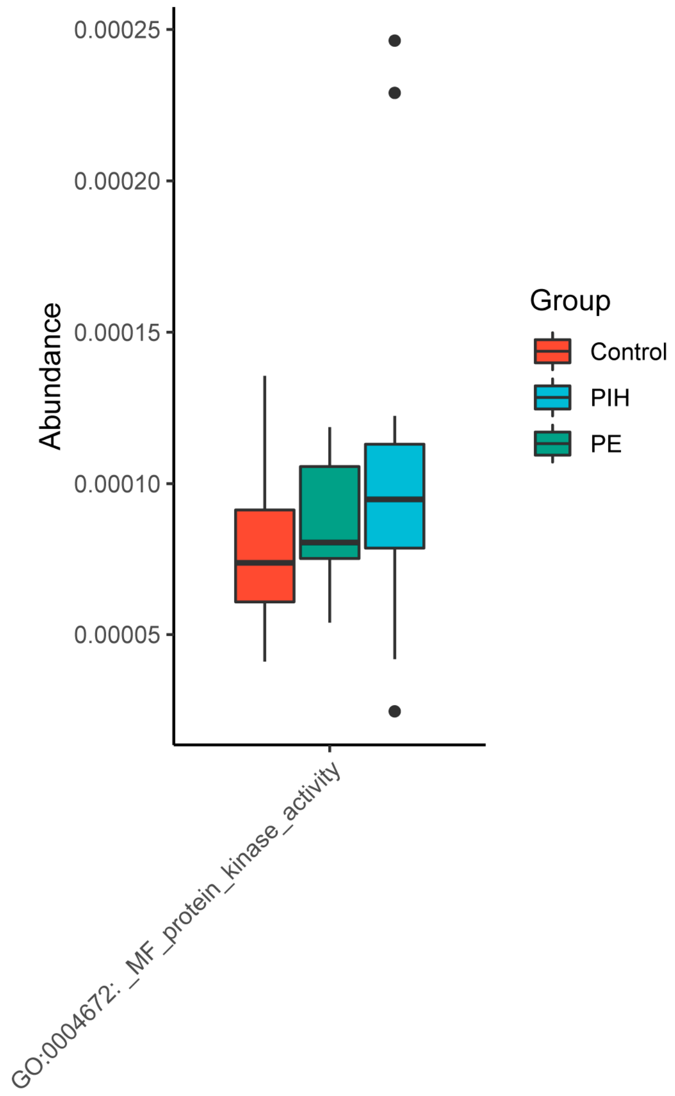

Figure S3. Boxplot of different function modules in three groups.

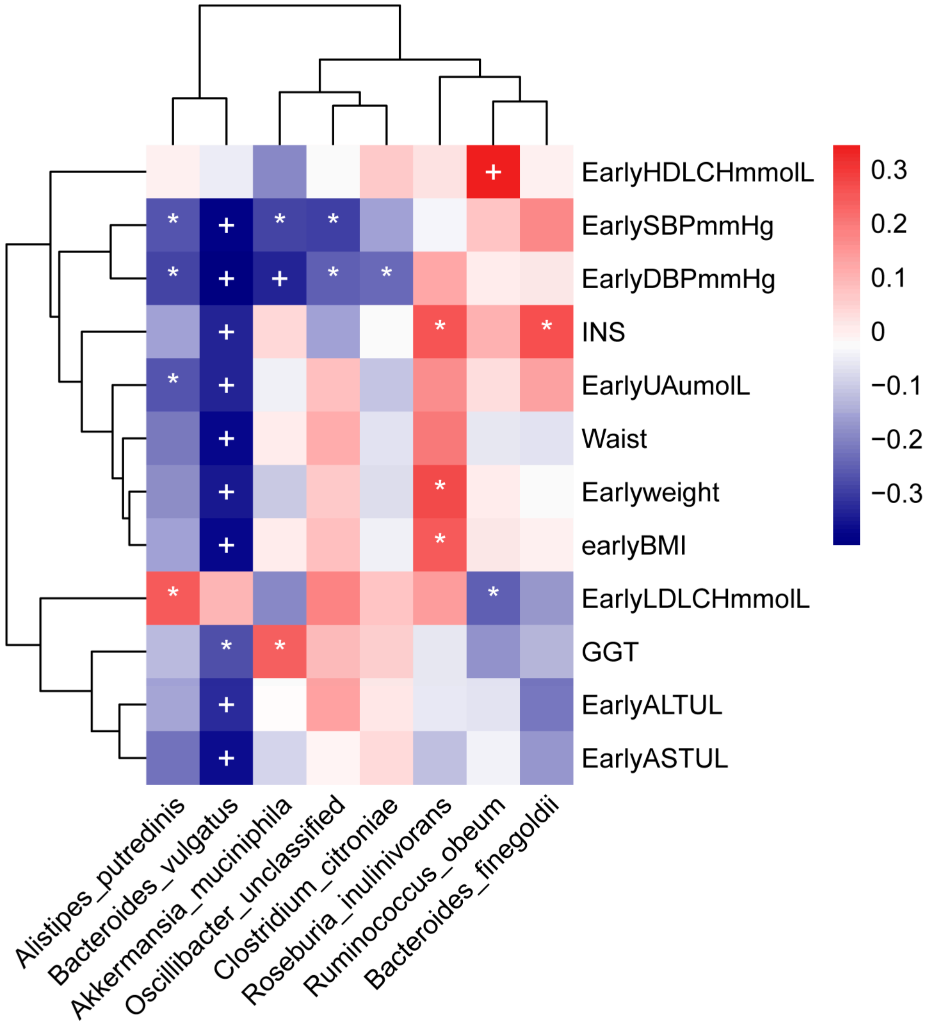

Figure S4. Heatmap of Spearman correlation between different species and clinical indices between three groups. Positive correlations are indicated in red text and negative correlations are indicated in blue text. (* $p < 0.05$; + $p < 0.01$)

HDLCH, high density lipoprotein cholesterol; SBP, systolic blood pressure; DBP, diastolic blood pressure; INS, insulin; UA, uric acid; BMI, body mass index; LDLCH, low density lipoprotein cholesterol; GGT, glutamyltransferase; AST, glutamic oxalacetic transaminase; ALT, glutamic pyruvic transaminase.

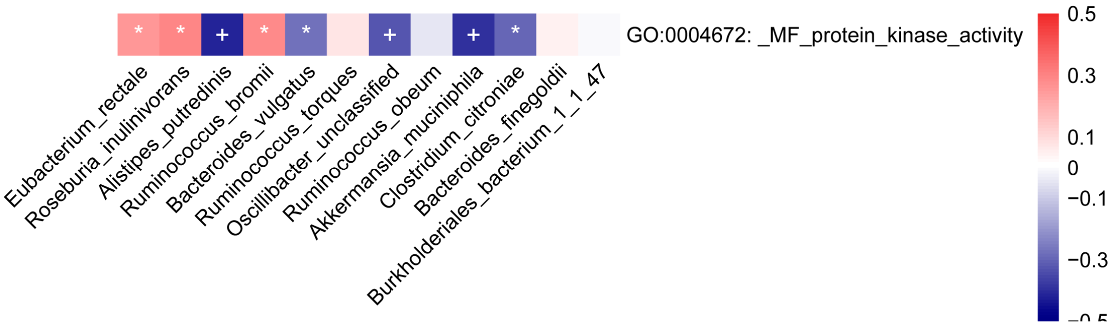

Figure S5. Heatmap of Spearman correlation between different species and function modules between three groups. Positive correlations are indicated in red text and negative correlations are indicated in blue text. (* $p < 0.05$; + $p < 0.01$)
